# *Nakaseomyces glabratus* (*Candida glabrata*) MLST Genotypes in Central Poland

**DOI:** 10.3390/ijms26094407

**Published:** 2025-05-06

**Authors:** Robert Kuthan

**Affiliations:** Department of Medical Microbiology, Medical University of Warsaw, 5 Chałubińskiego Street, 02-004 Warsaw, Poland; robert.kuthan@wum.edu.pl; Tel.: +48-22-628-27-39

**Keywords:** *Nakaseomyces glabratus*, *Candida glabrata*, molecular typing, MLST, drug susceptibility, Poland

## Abstract

*Nakaseomyces glabratus* is a medically important fungal pathogen responsible for various opportunistic, life-threatening, and fatal infections, mainly among immunodepressed patients worldwide. Herein, genotypes identified in Central Poland by multilocus sequence typing (MLST) are presented. Along with the genotyping, drug susceptibility was performed. The research was conducted on 30 non-redundant clinical strains, and 15 distinct sequence types (STs) were identified, including three novel STs: ST212, ST213, and ST214. The most prevalent sequence types were ST3, ST6, and ST10. Antifungal susceptibility testing revealed varied resistance rates to azoles, with fluconazole susceptibility at 16.7% and high susceptibility to amphotericin B. No correlation between ST and antifungals MIC were found. The study findings highlight the genetic diversity of *N. glabratus* in Central Poland and the role of surveillance and research to elucidate antifungals resistance and molecular epidemiology of *N. glabratus*.

## 1. Introduction

Unicellular fungi can exist in humans as both commensal microbiota and pathogens. As mycotic pathogens, they are primarily responsible for opportunistic infections with a wide range of clinical presentations, from mucosal infections and infections affecting the skin and nails to life-threatening organ and systemic candidiasis, particularly fungemia. The severity of infection is related to the infection localization, the virulence of a strain, and the patient’s immune status [1,2,3]. 

One such fungus is *Nakaseomyces glabratus* (previously *Candida glabrata*) classified in the order *Saccharomycetales* [4]. Currently, *N. glabratus* is recognized as the second most common etiological agent of fungemia, following *C. albicans*, particularly among immunocompromised individuals and patients undergoing intensive care treatment [5,6,7,8]. Moreover, the pathogenicity of *N. glabratus* is consistent with its low susceptibility to fluconazole and the rapid development of resistance to other antifungal drugs, particularly azoles such as voriconazole and echinocandins, which can occur even during treatment [5,9,10,11,12,13]. Resistance mainly arises from point mutations in specific genes. For example, mutations in *FKS1* (β-1,3-glucan synthase gene) confer resistance to echinocandins [14,15]. Resistance to azoles may result from overexpression of efflux pump genes (*CDR1*, *CDR2*, *SNQ2*) and from mutations in ergosterol biosynthesis genes—primarily *ERG11*, and in *ERG3*—often developing under anidulafungin exposure [16,17]. Once acquired, resistance in fungal strains can persist even without selective pressure. The stability of these resistance-conferring mutations under non-selective conditions has major epidemiological implications: resistant isolates can spread to other patients or the hospital environment, leading to clonal dissemination of resistant strains [18].

Formerly, *C. glabrata* was defined as a catch-all containing a complex of three different but closely related species: *C. glabrata sensu stricto*, plus two emerging cryptic species *C. nivariensis* and *C. bracarensis*, under the term *C. glabrata sensu lato* [19,20,21,22]. Identification relying on biochemical tests or phenotypic testing renders the distinguishing of these three species ineffective. Species differentiation within the complex may be achieved with molecular biology techniques or matrix-assisted laser desorption/ionization time-of-flight mass spectrometry (MALDI ToF MS) [21,23,24]. Phylogenetic studies have reclassified these under the new genus *Nakaseomyces* [25]. 

Infections due to the unicellular fungi classified as the genus *Candida* have been continuously and increasingly reported in recent years. As observed in numerous studies, *N. glabratus* prevalence in clinical settings varies within similar ranges, positively correlating with the length of a hospital stay (LOS) and specific niches, mainly occurring with the rectum, perianal area, and oropharynx [26,27,28]. Our previous research indicates that the isolation frequency of *N. glabratus* from clinical specimens ranges from 15.6% to 21.7% [29,30]. However, our observations of *N. glabratus* colonization of patients in a hospital in Warsaw are over two times lower (unpublished data). Since yeast infections are usually caused by the same fungi that colonize a patient, it is recommended to perform microbiological surveillance to identify patients at risk of fungal infection [31,32,33,34]. In the case of *N. glabratus*, similar to other *Candida* species, the transition from a colonizing to a pathogenic microbiota, as well as organ and/or bloodstream dissemination, is mediated by the injury of mucosal membranes, primarily due to the use of medical devices (cannulas, catheters) during surgery, intubation, and other invasive medical procedures [32]. It has recently been suggested that gastrointestinal microbiota has some involvement in the dissemination of *N. glabratus*. Studies validating this idea were performed on a mouse model. However, similar mechanisms may also occur in humans [35].

The genome of *N. glabratus* is composed of 13 chromosomes (Chr)—ChrA—ChrM, nuclear DNA, and mitochondrial DNA [36]. Molecular typing techniques, including random amplified polymorphic DNA (RAPD), restriction fragment length polymorphism (RFLP), pulsed-field gel electrophoresis (PFGE), multilocus sequence typing (MLST), microsatellite analysis, and whole genome sequencing (WGS), have revolutionized population, epidemiological, and evolutionary studies of human pathogens. The MLST genotyping method in particular, which consists of a comparative analysis of the sequence of fragments of genes responsible for basic metabolism, has become a reference method used for phylogenetic and epidemiological study of pathogenic microorganisms.

The MLST method is based on sequencing multiple loci of housekeeping genes and detecting single nucleotide polymorphisms (SNPs). Sequencing results reveal a unique combination of polymorphisms (allelic variation of each locus). These data sets are uploaded to a public database (public databases for molecular typing and microbial genome diversity (PubMLST), https://pubmlst.org) for molecular typing and to record microbial genome diversity. Based on the combination of the allelic variants of each gene, a tested isolate is assigned to a particular pre-existing sequence type (ST). In case of a new allele combination identification, the data curator creates and assigns a new ST number. The MLST typing of *C. glabrata* (later renamed *N. glabratus*) has been developed by Dodgson et al. [37]. The *N. glabratus* MLST typing is based on using the sequences of the internal fragments of six housekeeping genes, including: *FKS*—1,3-beta-glucan synthase (GenBank accession no. AF229171), *LEU2*—3-isopropylmalate dehydrogenase (GenBank accession no. U90626), *NMT1*—myristoyl-CoA, protein N-myristoyltransferase (GenBank accession no. AF073886), *TRP1*—phosphoribosylanthranilate isomerase (GenBank accession no. U31471), *UGP1*—UTP-glucose-1-phosphate uridylyltransferase (GenBank accession no. AB037186), and *URA3*—orotidine-5′-phosphate decarboxylase (GenBank accession no. L13661). These genes are located on chromosomes G, H, A, C, L, and I, respectively.

At the time of writing (March 2025), the *N. glabratus* MLST database (PubMLST https://pubmlst.org/organisms/candida-glabratashows; accessed on 15 March 2025) [38] contains a total number of 276 alleles sequences that are used for MLST profiling: *FKS* (44 alleles, fixed length 589 bp), *LEU2* (43 alleles, fixed length 512 bp), *NMT1* (68 alleles, fixed length 607 bp), *TRP1* (47 alleles, fixed length 419 bp), *UGP1* (34 alleles, fixed length 616 bp), *URA3* (40 alleles, fixed length 602 bp).

It should be noted that, despite more than 20 years since the initial description of the MLST typing scheme for *N. glabratus*, the number of isolates (2357) deposited in PubMLST is still relatively small compared to, for example, *Staphylococcus aureus*, *Pseudomonas aeruginosa*, or *C. albicans* (43,391; 10,576; 5805 isolates, respectively). This may affect the ability to assess the genetic diversity of *N. glabratus* worldwide. Relatively low numbers of these strains may be a consequence of the fact that a plethora of research is focused on the molecular mechanisms of antimycotic resistance. However, as more research is performed using the whole genome sequencing (WGS) technology, our understanding of *N. glabratus* genetic diversity, molecular aspects of its pathogenicity, and overall drug resistance will be improved. Indeed, next-generation sequencing (NGS) and WGS technology significantly advance our understanding of these aspects in the case of *N. glabratus* and other pathogenic fungi [39,40,41,42,43,44,45].

This study aimed to characterize the genetic diversity of *N. glabratus* isolates from Central Poland using MLST, determine the antifungal susceptibility profiles of “naïve” strains (isolates from patients with no prior antifungal exposure), and assess correlations between ST and antifungals minimum inhibitory concentrations. This study provides the first baseline information essential for future surveillance, outbreak detection, and molecular epidemiology.

## 2. Results

### 2.1. Genotyping 

**Figure 1:**
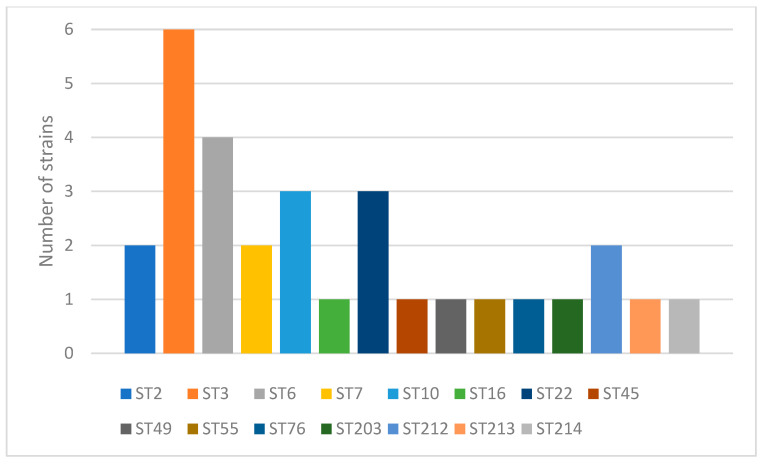
Identified sequence types (ST) and their occurrence. Each color bar represents an individual ST.

Genotyping was performed on 30 non-duplicate clinical isolates of N. glabratus. The strains had been isolated from patients, residents of Central Poland (Masovian Voivodeship), admitted to the hospital. Rectal swabs were collected from these patients on admission as part of routine epidemiological screening for colonization with pathogenic microorganisms. A total of 15 different sequence types were identified by the MLST method. The most prevalent were ST3 (n = 6; 20%), followed by ST6 (n = 4; 13.3%), and ST10 and ST22 (each n = 3; 10%). ST2 and ST7 have been identified in two strains each. In six strains, already described STs (ST16, ST45, ST49, ST55, ST76, and ST203) have been identified. The remaining four strains represented new STs. Based on their allelic variants, the *N. glabratus* database curator has assigned them a new ST number. These new STs are assigned as ST212 (2 strains), ST213 (1 strain), and ST214 (1 strain). The locus name and allele number of all tested strains are presented in Table 1. In Figure 1, all the identified STs and their occurrences are shown. 

Analysis of the allelic type of each locus shows higher diversity within the NMT1 locus with 12 allelic types, followed by LEU2 and TRP1, each with 11 allelic types. For the locus FKS, nine allelic types have been identified, and for URA3, seven allelic types have been identified. The lowest number of allelic types (5) was identified within the UGP1 locus. A detailed summary of the allelic types for each locus, along with their overall frequency of occurrence, is provided in Table 2.

### 2.2. Antimicrobial Susceptibility Testing

**Figure 2:**
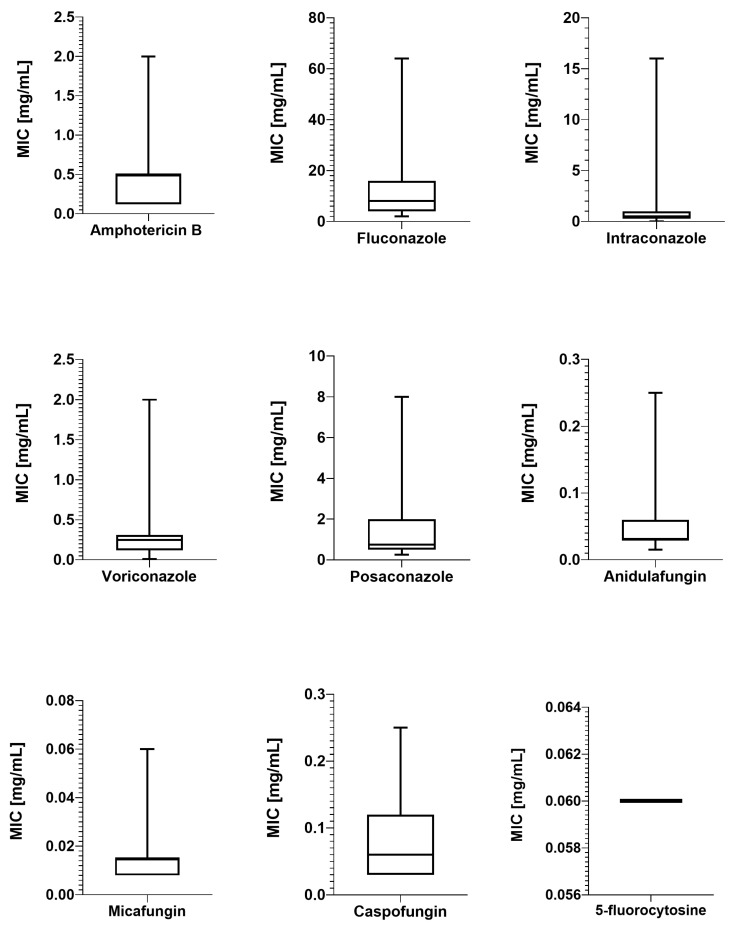
A box-and-whisker plot illustrating the distribution of MIC values. A horizontal line within each box indicates the median, while the whiskers represent the range from the minimum to the maximum observed values.

To obtain the susceptibility profile on naïve isolates, those neither originating from a hospital environment nor exposure-induced resistance, this study included patients with no history of prior hospitalization or antifungal treatment according to their medical history. In Figure 2. the distribution of MIC values is presented. Antimicrobial susceptibility testing (AST) revealed that 96.7% of tested isolates were susceptible to amphotericin B (AB) and, at the same time, classified as wildtype (WT) according to the Epidemiological Cut-Off Value (ECOFF) value (1 mg/L). 

The results of MICs determination for azoles were as follows: fluconazole (FZ) ranged from 2.0 to 64 mg/L; MICs of the remaining azoles—itraconazole (IZ) and voriconazole (VOR)—ranged from 0.015 to 16.0 mg/L and from 0.008 to 2.0 mg/L, respectively; and posaconazole (PZ) MIC values ranged from 0.25 to 8.0 mg/L. 

Based on the EUCAST Antifungal Clinical Breakpoint Table v. 11.0 [45], valid from 2 December 2024, interpretation values, the percentage of susceptibility for FLU was 16.7%, with 83.3% categorized as intermediate-susceptibility. For IZ, VOR, and PZ, the EUCAST guidelines do not provide an interpretation of the MICs. However, given the ECOFFs and the basis on which isolates are classified as wildtype or non-wildtype (non-WT), 28 were WT for IZ, and 29 were WT for VOR. In the case of PZ, 29 isolates were WTs.

In the case of echinocandins, the results for anidulafungin and micafungin were similar, with 96.7% being susceptible to these drugs. The strains were also classified as wildtypes. For caspofungin, the EUCAST provides neither interpretation for the MIC nor ECOFFs, and it is recommended that the susceptibility be interpreted based on the results of anidulafungin and micafungin. If a strain is susceptible to both of them, it is also susceptible to caspofungin. Therefore, two isolates should be interpreted as resistant to caspofungin since each was resistant to one of the aforementioned drugs. As for 5-fluorocytosine (FC), the lack of ECOFF for caspofungin makes it impossible to assign them as WT or non-WT.

The drug susceptibility results for all strains tested are presented in the Supplementary Materials (Table S1).

### 2.3. Correlation Between STs and MICs

**Figure 3:**
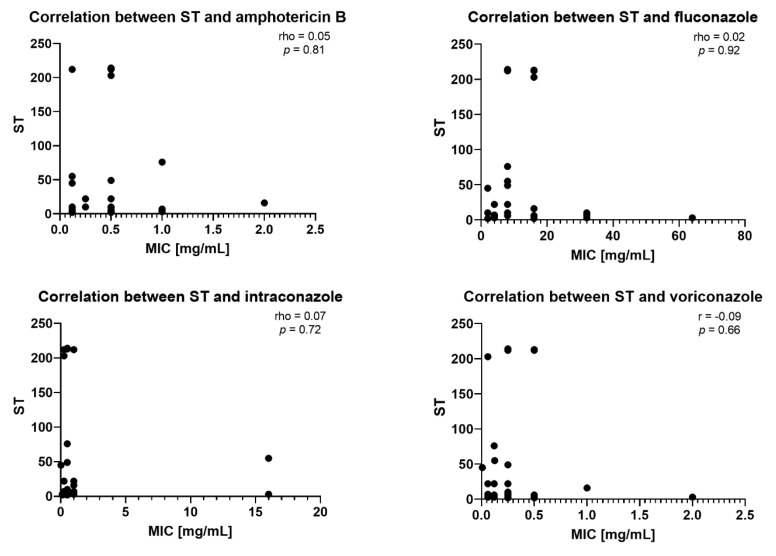

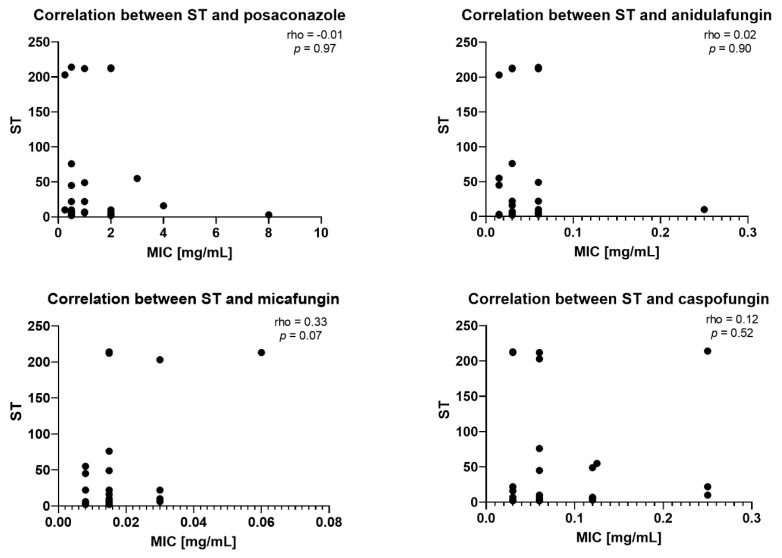
Scatter-plot analysis of Spearman’s rank correlation coefficient of ST with antifungal minimum inhibitory concentrations (MICs). Each dot represents a single clinical isolate. Legend: X-axis: MIC in mg/mL; Y-axis: ST—sequence type. *p* value < 0.05 is considered statistically significant. Spearman’s correlation coefficient is signified by rho.

Spearman’s rank correlation coefficient (rho) was used to examine the relationship between sequence type (ST) and minimum inhibitory concentration (MIC) values of antifungals. The analysis revealed uniformly weak and non-significant Spearman correlations across all antifungals, with rho values of 0.05 (*p* = 0.81) for amphotericin B; 0.02 (*p* = 0.92) for fluconazole; 0.07 (*p* = 0.72) for itraconazole; 0.09 (*p* = 0.66) for voriconazole; −0.01 (*p* = 0.97) for posaconazole; 0.02 (*p* = 0.90) for anidulafungin; 0.12 (*p* = 0.52) for caspofungin, and a borderline trend with micafungin (rho = 0.33, *p* = 0.07), indicating that ST is essentially independent of the susceptibility profiles of polyenes, azoles, and most echinocandins, with only a non-significant tendency toward co-variation with micafungin MICs. As the MIC value for 5-fluorocytosine was 0.06 mg/mL in all 30 isolates tested (no variability at all), calculating the Spearman’s rank correlation coefficient for this pair of variables was impossible. Figure 3 presents eight scatterplots for the correlation between ST and antifungal MIC.

**Figure 4:**
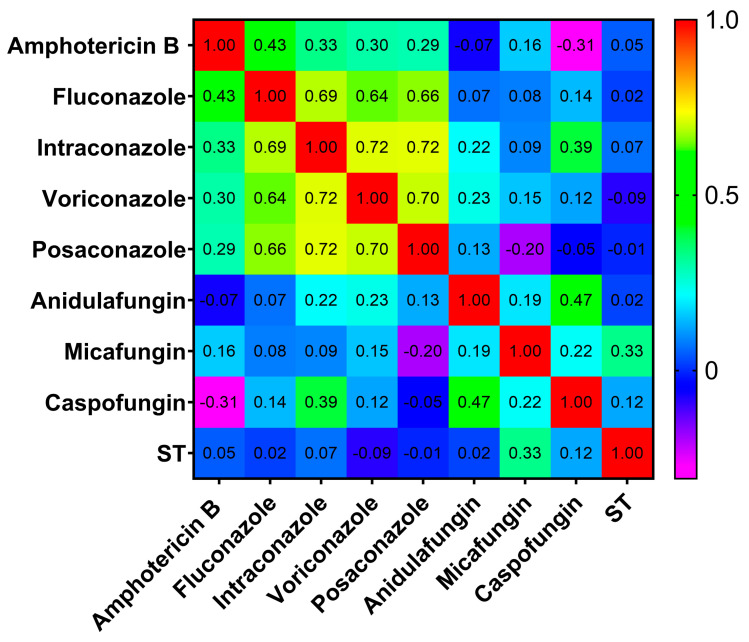
Heatmap of Spearman’s correlation between STs and amphotericin B, fluconazole, itraconazole, voriconazole, posaconazole, anidulafungin, micafungin, and caspofungin, where the color bar indicates rho = +1 (red) as perfect positive correlation; 0 < rho < 1 (yellow/green) as the two variables tending to increase or decrease together; rho = 0 (cyan) as no association; −1 < rho < 0 (blue/purple) as one variable increasing while the other decreases; and rho = −1 (magenta) as perfect negative (inverse) correlation.

Figure 4 presents a heat map illustrating the correlation between antifungals and STs. 

## 3. Discussion

A steady increase in fungal infections negatively affecting patients’ health and even leading to life-threatening conditions is being observed worldwide [46,47]. For this reason, mycoses require early diagnosis and effective treatment. Culture-based infection etiology identification is not always successful. Even when a microorganism is cultured, distinguishing colonization from actual infection may be challenging for both the medical microbiologist and the clinicians. A growing resistance to antifungal drugs further complicates the treatment and outcomes of fungal infections. 

*N. glabratus* is a natural component of the human mycobiota, colonizing various body niches. Moreover, it is also a significant pathogen, responsible for opportunistic and healthcare-associated infections, mainly bloodstream infections (BSIs) [48]. The gastrointestinal tract is considered one of the primary body niches from which fungus cells may disseminate to the circulatory system and organs. Infections originating from the gastrointestinal tract via this route are widely documented [49,50,51,52,53,54]. Research by Chew et al. [55] demonstrated that *N. glabratus* was among the leading causes of fungal BSIs, accounting for 28.2% of all isolates cultured from blood cultures in Singapore between 2018 and 2021. 

However, retrospective studies from some countries indicate that other *Candida* species are the leaders of bacteremia. For example, a retrospective study conducted in a teaching hospital in China from 2006 to 2024 showed that the predominant species isolated were *C. albicans* (31%), *C. parapsilosis* (20.09%), and *C. tropicalis* (18.38%), with an average isolation rate of 13.25% for *N. glabratus* identified [27]. Similarly, in a tertiary hospital in Greece, a study of bacteremia etiology from 2020 to 2024 showed that *C. albicans* prevalence in positive blood cultures has dropped, demonstrating an average value of 23.68% during the study period. Meanwhile, the incidence of bacteremia caused by *C. parapsilosis* has risen, with the average rate being 52.42%, and the highest value (61.3%) was recorded in 2022. Additionally, since 2022, *C. auris* isolation from candidemia cases had an average value of 20.4%, yet the relative contribution of *N. glabratus* was only 6.3% [56].

These findings undoubtedly indicate the increasing prevalence of non-albicans *Candida* in BSIs. The reasons for the rising incidence of infections caused by *N. glabratus* and other unicellular fungi, mainly of the genus *Candida*, are complex and not yet fully understood. They may be related to geographical factors, patient health profiles, the availability and quality of microbiological diagnostics, the pharmacotherapy standards applied empirically and therapeutically in these regions, or hygienic procedures and other anti-infection measures undertaken in differing hospital settings.

Another consideration is the roles and dynamics demonstrated between bacteria and fungi colonizing the gastrointestinal tract [57]. Antibiotic use and its impact on intestinal dysbiosis may influence and even amplify the dissemination of *N. glabratus* [58,59]. In mouse models, anaerobic bacteria (*Faecalibaculum rodentium* and *Enterococcus faecalis*) were demonstrated to promote *N. glabratus* dissemination to the organs but not colonization of the gastrointestinal tract [35]. The results of these studies indicate that this process may happen among humans. Moreover, it has been indicated that antibiotics not only influence the bacterial microbiota of the gastrointestinal tract but also indirectly affect fungal microbiota, underlying the complexity of interactions between bacteria and fungi.

In 2022, the WHO published a list of fungal priority pathogens, which included *N. glabratus*, *C. parapsilosis*, *C. tropicalis*, and four other pathogenic fungi in the High Priority category. The inclusion of *N. glabratus* in this group highlights the need for increased research into its genetic diversity and drug resistance, increased surveillance, and the development of newer diagnostics [60].

Despite numerous epidemiological and microbiological studies analyzing the correlations between the genotype and drug resistance profile of *N. glabratus*, no definitive relationships have been established. So far, genotyping of *N. glabratus* strains isolated from patients in Poland has been performed primarily by random amplified polymorphic DNA (RAPD) PCR [61,62,63,64] but not by MLST. To our knowledge, this study provides, for the first time, insight into the occurrence of *N. glabratus* sequence types (STs) isolated from patients in Poland, specifically in its central region. 

Numerous molecular typing methods (RFLP, PFGE, MLST, satellite typing) have varying discriminatory power, cost, effort, and technical reproducibility. In terms of a possible comparison of isolates that do not necessarily occur within a small pool (e.g., originating from an outbreak within a ward and/or a hospital), but between different places of origin, with various clinical materials, and over a wider period, MLST typing is a relatively inexpensive method that can be deployed and performed quickly. The most important thing about the MLST typing method is that its results allow the comparison of strains over both time and place.

Over the following years (2016–2025), the number of newly added isolates to the PubMLST database that were subjected to MLST typing gradually and significantly increased, with a median of 251 isolates per year and a range of 4 to 889 isolates per year (until the end of February 2025), and currently equals 2357 isolates. These isolates originate from 6 continents and represent 33 countries. 

### Comparison of Poland to the World STs

The highest number of strains typed by MLST comes from five countries: China—489, USA—717, Spain—130, UK—120, and Denmark—103. Taken together, isolates from these countries account for 66.14% of the total strains typed by MLST, with the USA contributing the largest share (30.42%), followed by China (20.75%), Spain (5.52%), the UK (5.09%), and Denmark (4.37%). Poland, with 30 isolates typed, has a contribution rate of 1.27%. The five most prevalent types in those countries are the following STs, where the number of isolates is given in parentheses: China—ST7 (236), ST19 (36), ST45 (25), ST10 (21), ST20 (15); USA—ST16 (119), ST3 (100), ST19 (98), 10 (59), 15 (54); Spain—ST3 (64), ST10 (10), ST149 (9), ST34 (8), ST 6 (7); UK—ST3 (19), ST6 (13), ST10 (10), ST2 (9), ST8 (7); and Denmark—ST3 (19), ST6, and ST22 (9 strains each), ST19 (8), ST8 (7). In Poland, the most common STs were ST22 (3), ST6 (4), ST10 (3), ST3 (6), and ST2 and ST7 (2 strains each). Among the mentioned countries, several STs were occurring only within their regions, with the highest number of unique types reported in China, 37 STs, followed by the USA with 33 STs. Among the remaining countries, the UK had 14 unique STs; Denmark—11; Spain—3 (ST14, ST146, ST150); and Poland—2 (ST213, ST214).

Remarkably, of all the STs deposited in the PubMLST database, 167 STs were detected only once. For example, STs 117, 114, 115, 116, and 117, which have so far been reported in Tanzania only [65].

Unlike the worldwide distributed STs, several sequence types are present only in some regions. Thus, whether these sequence types are indeed endemic or are possibly endemic remains to be elucidated. Of the defined STs, those occurring more than once (a unique ST with two or more isolates) were counted to be 45 STs: ST11, ST20, ST21, ST30, ST40, ST67, ST95, ST113, ST115, ST117, ST118, ST120, ST123, ST135, ST136, ST137, ST139, ST140, ST149, ST151, ST152, ST153, ST154, ST166, ST169, ST187, ST197, ST204, ST210, ST212, ST216, ST218, ST234, ST242, ST244, ST255, ST256, ST257, ST283, ST286, ST292, ST295, ST297, ST303, ST307. These isolates were reported from 19 countries: Australia, Brazil, China, Denmark, France, India, Iran, Japan, Kuwait, Namibia, Norway, Poland, Qatar, South Korea, Spain, Taiwan, Tanzania, the UK, and the USA [37,65,66,67,68,69,70,71,72,73,74,75,76,77,78,79].

Among the unique STs, those with the highest number of isolates are ST139 from South Korea, which was identified among six isolates, ST210 from the USA, which was found in nine isolates, and nine isolates from Spain belonging to ST149 [73].

The European countries from which isolates with determined ST are described (based on the data from the PubMLST database) are Austria, Belgium, Denmark, France, Germany, Italy, Norway, Poland, Portugal, Spain, Switzerland, the Netherlands, and the UK. They represent 79 STs (20.18% of total STs). 

Among the established STs, ST3–the second most common ST globally, constituting 13.8% of the examined strains on a global scale—was the most prevalent in Poland (0.25%). Values given after a country name refer to prevalence on a worldwide scale. The ST3 has been also identified in Africa (Tanzania, 0.04%), Asia (China, 0.85%; Iran, 0.38%; Japan, 0.08%; Kuwait, 0.30%; Qatar, 0.55%, Taiwan, 0.25%), Europe (Austria, 0.13%; Belgium, 0.17%; Denmark, 0.81%; France, 0.47%; Germany, 0.25%; Portugal, 0.04%; Spain, 2.72%; the Netherlands, 0.04%; the UK, 0.81%), North America (Mexico, 0.04%, USA, 4.24%), Oceania (Australia, 0.34%), and South America (Argentina and Brazil, each 0.38%; Chile and Colombia, each 0.13%; Ecuador, n = 0.04%).

The second-most prevalent (0.08%) ST in Poland was ST7, which has 15.8% on a global scale, thus being the most common ST worldwide. This ST is notably more common in Asian countries: China, 10.01%; Iran, 0.59%; Japan, 1.32%; Kuwait, 0.17%; Qatar, 0.51%; South Korea, 0.04%; Taiwan, 0.55%; and Thailand, 0.08%. The ST7 in Africa has been identified in Tanzania only, at 0.13%. In Europe, ST7 has been identified in the following countries: Austria, 0.17%; Belgium, 0.13%; Denmark, 0.21%; France, 0.25%; Germany, 0.08%; Spain, 0.08%; the UK, 0.25%. In the USA, the number of strains belonging to ST7 is 15 (0.64% on the global scale). In Australia, there are five strains (0.21%). In South America, ST7 has been identified in Brazil at 0.17% and Ecuador at 0.04%.

The other STs (2, 6, 10, 16, 22) identified in Poland were variably distributed worldwide, with ST16 occurring most often in the USA, at 5.05%. 

Several STs have been detected in Poland with only one isolate (0.04%) each, such as ST45, ST49, ST55, ST76, and ST203. These ST variants are relatively rare globally and may be specific to certain regions or have emerged more recently. ST55 constitutes 2.2% on a global scale: in Africa (Namibia, 0.17%), in Asia (China, 0.51%; Kuwait, 0.25%; Taiwan, 0.42%, Thailand, 0.25%), in Europe (Denmark, 0.08%; France, 0.21%; the UK, 0.13%), and in North America (USA) and Oceania (Australia) in each region at 0.08%. ST45 comprises a lower global prevalence, at 1.4%. It has also been reported in Asia (China, 1.06%), Europe (Austria, 0.04%), North America (the USA, 0.17%), and Oceania (Australia, 0.04%). ST76 is exceedingly rare globally at 0.31% and has been identified in Austria (0.08%), and the USA (0.13%). Lastly, ST49 and ST203 have a very small global prevalence, at 0.1% and 0.2%, respectively. Outside of Poland, ST49 has been identified only once (0.04%) in the USA. Of equal rarity, ST203 has appeared only in China four times (0.17% on a global scale). 

The new STs, specifically ST212, ST213, and ST214, accounted for 0.1% (2 isolates), 0.05% (1 isolate), and 0.05% (1 isolate), respectively. These STs have not been identified in other countries, indicating a unique genetic diversity in the Polish population of *N. glabratus*.

This present study has unveiled a notable diversity of STs within Poland, identifying three previously unreported STs: ST212, ST213, and ST214. Remarkably, as per the available data in the PubMLST database, these STs have not been documented in any other country [38]. It is worth noting that no studies on the genetic diversity of *N. glabratus* have been conducted using the MLST technique in any country but Germany, neighboring Poland, as well as in other areas of Central and Eastern Europe and the Balkan Peninsula. Thus, the landscape of STs in Europe may not reflect the accurate distribution of STs on this continent. 

Further research may show a similarity, as is the case of *C. albicans*, that the prevalence of STs depends on geography and ethnicity. This diversity can be influenced by globalization, particularly increased global migration and tourism. It is increasingly likely that in the near future, we will observe a clearer picture of the distribution of sequential types and clonal complexes and clades in the world [80,81].

Meng et al. [82] observed that among 122 isolates collected from the oral cavity, female reproductive tract, and environment, ST7 and ST45 were more prevalent in the oral cavity samples (32.1% and 32%, respectively). In the authors’ opinion, the observed distribution may be attributed to the small number of specimens analyzed. The same study found that the colonization rate is not related to age. Among patients who had no previous antifungal treatment, resistance of *N. glabratus* to fluconazole was found to be at the rate of 10.23%, a very high rate (79.53%) to itraconazole, and definitely lower to voriconazole and amphotericin B, with 5.17%, and 0.47%, respectively. The described high rate of drug-resistant isolates to itraconazole is relatively unusual since, as mentioned before, most infections caused by *N. glabratus* have an endogenous origin. Whether this situation is population- or region-specific remains to be elucidated. Other studies [83,84] indicate that the colonization rate and infection prevalence are variable among populations, investigated niches, and patients’ health conditions. In Ibero-American countries, the resistance to itraconazole has been reported to be 14% [85]. In the USA, itraconazole non-wildtype isolates were reported at 10.1% [86]. In Northeastern Iran, the resistance to itraconazole rate was reported at 3.6% [87]. Given the clinical and epidemiological importance of drug resistance and the genetic diversity of *N. glabratus*, it is crucial to determine whether specific sequence types (STs) exhibit higher levels of antifungal resistance. We therefore evaluated the correlation between antifungal MIC values and STs. Spearman’s rank correlation analysis revealed weak, non-significant associations for all drug classes except micafungin, for which a borderline trend toward correlation was observed. These findings suggest that ST is mainly independent of susceptibility to polyenes, azoles, and most echinocandins, with only a non-significant tendency toward co-variation with micafungin MICs. Our results agree with other reports [45,88,89,90]. In contrast, a study from China [90] reported a correlation between ST7 and ST10 and azoles resistance. Moreover, ST7 and ST15 (azole-susceptible) were independently associated with increased 30-day mortality. Similarly, in Korea, ST7 and ST3 predominated among bloodstream isolates and were linked to significantly higher 30-day mortality than other STs, no association between ST and fluconazole resistance was observed [66,91]. In Tanzania, ST18 demonstrated reduced fluconazole susceptibility [65]. In conclusion, these studies suggest that sequence type may correlate with antifungal resistance and clinical outcomes. In contrast, our analysis of Central Poland isolates revealed no significant association between ST3, ST7, or ST10 and azole MICs. These regional differences display the need for further research. Comparative analyses of isolates from invasive infections and colonized patients may help fully reveal the relationship between sequence type, drug resistance, and patient outcomes.

The presented data indicate the high genetic diversity within the *N. glabratus* species. At the same time, concerning currently available data (number of MLST typed isolates), the observed diversity may indicate probable geographic clustering or region-specific strain evolution. This statement is supported by the findings of several research papers presenting data from molecular studies and bioinformatic analyses of genetic relatedness between investigated strains [40,44].

## 4. Materials and Methods

### 4.1. Clinical Strains and Identification

Strains isolated from patients admitted to a large teaching hospital in Central Poland were used in this study. Anal swabs were taken from the patients as part of a routine screening for the colonization of MDR pathogen strains. Swabs for fungal cultures were inoculated onto Sabouraud agar supplemented with gentamicin and chloramphenicol (bioMérieux, Poland) and incubated at 30 °C for 48–72 h. Subsequently, individual colonies were isolated onto Sabouraud dextrose agar and incubated for 24–48 h. Identification was performed using MALDI-TOF MS, and strains identified as *N. glabratus* were used for further investigations. 

### 4.2. Genomic DNA Isolation 

For DNA extraction, 3.0 mL of overnight culture in a liquid Sabouraud medium was centrifuged and washed twice with sterile phosphate-buffered saline (PBS). The obtained pellets were then weighed to not exceed 100 mg wet mass (the isolation capacity of the DNA isolation kit). For DNA isolation, the GeneMATRIX Gram Plus & Yeast Genomic DNA Purification Kit (EURx, Gdańsk, Poland) was used. DNA isolation was performed according to the manufacturer’s specifications. The DNA purity and concentration were then analyzed with 260/280 nm UV–Vis absorbance measurements using the NanoDrop ND-1000 spectrophotometer (Thermo Scientific™, Waltham, MA, USA). DNA was also quantified using 1% agarose gel electrophoresis at a potential of 100 V (Consort EV265 Range Power Supply, Alpha Metrix Biotech, Rödermark, Germany). After separation, the DNA isolate was visualized under UV light after staining with a fluorescent SYBR^®^ green gel dye (Infinity VX2, Vilber Lourmat, Collégien, France).

To confirm the authenticity of MALDI-TOF MS results, the polymerase chain reaction (PCR) method, according to Kurtzman, was applied [92]. The internal transcribed spacer 1 (ITS-1), which characterizes *N. glabratus sensu stricto*, was identified using PCR. The NL-1 (5′-GCAT ATCAATAAGCGGAGGAAAAG’) and NL-4 (5′-GGT CCGTGTTTCAAGACGG’) primers were used for the amplification. The amplification was performed on a LabCycler (SensQuest, Goettingen, Germany) under the following conditions: 94 °C for 10 min for initial denaturation, followed by 30 cycles of denaturation at 94 °C for 30 s, then annealing at 50 °C for 1 min, performing initial elongation at 72 °C for 30 s, and then final elongation at 72 °C for 10 min. As a result, the identification of all tested strains as *N. glabratus sensu stricto* had been confirmed.

### 4.3. Genotyping by MLST

Six housekeeping gene loci (*FKS, LEU2, NMT1, TRP1, UGP1*, and *URA3*) were studied for all isolates as previously described [37]. Table 3 presents the target gene name, primer sequences, and other details.

The PCR reaction mixtures contained 1 μL (25 ng) of DNA, 0.5 μL of each primer (forward and reverse) at the concentration of 10 pM/µL, 2 μL of dNTP mix (premixed aqueous solutions of dATP, dCTP, dGTP, and dTTP, each at a final concentration of 2 mM), 4 μL of PCR 10× buffer, 1.3 μL of 25 mM MgCl2, 2.5 U of Taq DNA polymerase, 1μL DMSO, and PCR-grade H_2_0 to reach a final volume of 20 μL.

PCR conditions were optimized by temperature gradient PCR on the LabCycler (SensQuest, Goettingen, Germany). Then, the following reaction conditions were used: 94 °C for 10 min for initial denaturation, followed by 30 cycles of denaturation at 94 °C for 30 s, then annealing at 51 °C for 1 min, performing initial elongation at 72 °C for 1 min, and then final elongation at 72 °C for 10 min—all for *FKS*, *LEU2*, *NMT1*, *TRP1*, *UGP1*, and *URA3*. 

After the PCR reaction, the presence of a specific PCR product was screened by agarose gel electrophoresis. A sample of 4 μL was separated on 2% agarose with Green DNA Gel Stain, in electrophoresis conditions of 80 V/20min, and was finally visualized in UV light. The DNA sequences were then compared to the *N. glabratus* entries on the MLST database (PubMLST) [38].

### 4.4. Sample Preparation Sequencing

Amplified PCR products were enzymatically cleaned (hydrolysis of primers excess and nucleotides) by treatment with 1.1 μL Exonuclease I (Exo I) and 1.1 μL thermosensitive alkaline phosphatase FastAP™ (ThermoFisher, Waltham, MA, USA). Clean-up procedures were performed in the PCR tubes. Probes were then incubated in a PCR machine at 37 °C for 30 min, followed by incubation at 80 °C to inactivate enzymes completely.

Next, samples were labeled for sequencing. The reaction was set in two tubes: one for sequencing on the sense strand and the other for the antisense strand. The reaction mixture was composed of 1 μL of BigDye Terminator v1.1/v3.1 Sequencing Buffer (Applied Biosystems™, Waltham, MA, USA), 3 μL buffer, and 2 μL of 10× diluted primers (final concentration 1 pM/µL) used for the PCR (primer R for sequencing on the antisense strand, and primer F for sequencing on the antisense strand). PCR conditions at this step were as follows: stage 1—denaturation at 95 °C for 5 min; stage 2—amplification with 45 cycles at 95 °C for 30 s, then 50 °C for 20 s and 60 °C for 4 min; and lastly stage 3–4 °C for indefinite hold.

After labeling, the PCR products were subjected to purification by filtration. In this step, the PCR products were transferred onto the surface of Sephadex 50G and centrifuged for 2 min at a weight of 780 g. Filtrates were then collected onto a plate with 10 μL of formamide (Thermo Scientific, Waltham, MA, USA). A rubber cover was placed onto the plate, which was then inserted into the sequencing capillary of the Sanger sequencing machine—3130xL Genetic Analyzer (Applied Biosystems, Waltham, MA, USA). Sanger sequencing was performed on a 3130xL Genetic Analyzer (Applied Biosystems, Waltham, MA, USA). The results of sequencing were analyzed by Variant Reporter Software v2.0 (Applied Biosystems, USA) and Sequencing Analysis (Applied Biosystems, Waltham, MA, USA) and then underwent database analysis. The sequences obtained from Sanger sequencing were then aligned to the NT database with the NCBI BLAST online software version 2.12.0. DNA sequences obtained were compared to the MLST database (PubMLST) entries of *N. glabratus*.

### 4.5. Drug Susceptibility Testing

Drug susceptibility was determined by a colorimetric microbroth dilution test using Sensititre YeastOne YO10 AST Plate (ThermoFisherScientific, Waltham, MA, USA) according to the manufacturer’s instructions. The following drugs were tested: amphotericin B (AMB), fluconazole (FZ), voriconazole (VOR), itraconazole (ITR), anidulafungin (AND), micafungin (MF), caspofungin (CAS), posaconazole (PZ), and 5-fluorocytosine (FC), with the dilution range [µg/mL] as follows: AB, 0.12–8; FLU, 0.12–256; IZ 0.015–16; VOR, 0.008–8; POZ, 0.008–8; AND, 0.015–8; MYC, 0.008–8; CAS, 0.008–8; and 5FC, 0.06–64. For quality control of the MIC system, the following culture(s) from the American Type Culture Collection (ATCC) were used: *Candida krusei* ATCC 6258 and *Candida parapsilosis* ATCC 22019. For interpretation, the European Committee on Antimicrobial Susceptibility Testing (EUCAST) guidelines were used [93].

### 4.6. Statistical Analysis

The normality of the data distribution was assessed using the Shapiro–Wilk test. Spearman’s rank correlation coefficient (rho) was used to examine the relationship between bacterial sequence type (ST) and minimum inhibitory concentration (MIC) values of antifungals. Results were considered statistically significant at a *p*-value threshold of <0.05. Spearman’s rho was employed to quantify the strength and direction of associations between sequence type and antimicrobial susceptibility profiles. 

All statistical analyses, including data visualization and correlation computations, were conducted using GraphPad Prism software, version 8.0 (GraphPad Software, San Diego, CA, USA).

### 4.7. Phylogenetic Analysis

Phylogenetic analysis was conducted using the Maximum Likelihood method in MEGA12 [94], based on the concatenated sequence data of 30 *N. glabratus* isolates that were MLS typed in this study. A phylogenetic tree was then constructed to elucidate the evolutionary relationships among these isolates.

## 5. Conclusions

Our findings show that identifying three new sequence types (STs) in Poland highlights the need for further research. Studies involving a larger collection of *N. glabratus* isolates are necessary to provide a broader overview of the incidence and potential diversity of STs in Poland. These studies will also determine whether the STs found in Poland are similar to or different from those identified in Europe and worldwide, whether they are genetically diverse, and whether there is a correlation between ST and drug resistance profiles. This study found no correlation between ST and antifungal MIC values; however, further investigation is needed to explore potential correlations between ST and drug susceptibility. Our data may represent a “global niche” for these particular ST variants or reflect a lack of oversight in identifying these variants in other geographic regions, as there is significant diversity in ST variants, particularly across geographic locations such as Europe and North America, which are examples of regions with high ST diversity.

## Figures and Tables

**Table 1:** Sequence types and allele numbers of individual loci.

ID in the PubMLST Database	Genotype						Sequence Type
	*FKS*	*LEU2*	*NMT1*	*TRP1*	*UGP1*	*URA3*	
1936	1	2	2	1	1	1	2
1937	1	2	2	1	1	1	2
1914	5	7	8	7	3	6	3
1915	5	7	8	7	3	6	3
1924	5	7	8	7	3	6	3
1925	5	7	8	7	3	6	3
1926	5	7	8	7	3	6	3
1939	5	7	8	7	3	6	3
1919	2	5	7	5	1	2	6
1930	2	5	7	5	1	2	6
1931	2	5	7	5	1	2	6
1932	2	5	7	5	1	2	6
1917	3	4	4	3	3	4	7
1927	3	4	4	3	3	4	7
1928	8	4	3	5	1	2	10
1929	8	4	3	5	1	2	10
1933	8	4	3	5	1	2	10
1916	7	7	11	10	5	9	16
1918	7	5	6	12	1	8	22
1920	7	5	6	12	1	8	22
1922	7	5	6	12	1	8	22
1934	7	9	26	17	9	2	45
1921	6	6	29	2	3	4	49
1938	3	6	22	2	3	9	55
1923	10	14	12	11	3	2	76
1935	7	33	6	12	1	29	203
1383	1	23	2	1	1	1	212
1384	1	23	2	1	1	1	212
1385	20	13	21	9	3	2	213
1386	3	21	26	13	17	4	214

ID in the PubMLST database—identification number in the PubMLST database, *FKS*—1,3-beta-glucan synthase, *LEU2*—3-isopropylmalate dehydrogenase, *NMT1*—myristoyl-CoA, protein N-myristoyltransferase, *TRP1*—phosphoribosylanthranilate isomerase, *UGP1*—UTP-glucose-1-phosphate uridylyltransferase, *URA3—*orotidine-5′-phosphate decarboxylase.

**Table 2:** Allelic types of individual loci and their overall frequency of occurrence.

*FKS*		*LEU2*		*NMT1*		*TRP1*		*UGP1*		*URA3*	
Allelic Type	Number of Incidence	Allelic Type	Number of Incidence	Allelic Type	Number of Incidence	Allelic Type	Number of Incidence	Allelic Type	Number of Incidence	Allelic Type	Number of Incidence
1	4	2	2	2	4	1	4	1	16	1	4
2	4	4	5	3	3	2	2	3	12	2	10
3	4	5	7	4	2	3	2	5	1	4	4
5	6	6	2	6	4	5	7	9	1	6	6
6	1	7	7	7	4	7	6	17	1	8	3
7	6	9	1	8	6	9	1			9	2
8	3	13	1	11	1	10	1			29	1
10	1	14	1	12	1	11	1				
20	1	21	1	21	1	12	4				
		23	2	22	1	13	1				
		33	1	26	2	17	1				
				29	1						

*FKS*—1,3-beta-glucan synthase, *LEU2*—3-isopropylmalate dehydrogenase, *NMT1*—myristoyl-CoA, protein N-myristoyltransferase, *TRP1*—phosphoribosylanthranilate isomerase, *UGP1*—UTP-glucose-1-phosphate uridylyltransferase, *URA3*—orotidine-5′-phosphate decarboxylase.

**Table 3:** The characteristics of the PCR primers used for MLST typing.

Primer Name	Primer Sequence	Target Gene	Concentration for PCR	Concentration for Sanger Sequencing	Primer Length [nt]	Product Size [bp]	Reference
FKS-F	GTCAAATGCCACAACAACAACCT	1,3-beta-glucan synthase	10 pM/µL	1 pM/µL	23	589	[37]
FKS-R	AGCACTTCAGCAGCGTCTTCAG		10 pM/µL	1 pM/µL	22		
LEU2-F	TTTCTTGTATCCTCCCATTGTTCA	3-isopropylmalate dehydrogenase	10 pM/µL	1 pM/µL	24	512	
LEU2-R	ATAGGTAAAGGTGGGTTGTGTTGC		10 pM/µL	1 pM/µL	24		
NMT1-F	GCCGGTGTGGTGTTGCCTGCTC	myristoyl-CoA, protein N-myristoyltransferasea	10 pM/µL	1 pM/µL	22	607	
NMT1-R	CGTTACTGCGGTGCTCGGTGTCG		10 pM/µL	1 pM/µL	23		
TRP1-F	AATTGTTCCAGCGTTTTTGT	phosphoribosyl-anthranilate isomerase	10 pM/µL	1 pM/µL	20	419	
TRP1-R	GACCAGTCCAGCTCTTTCAC		10 pM/µL	1 pM/µL	20		
UGP1-F	TTTCAACACCGACAAGGACACAGA	UTP-glucose-1-phosphate uridylyltransferase	10 pM/µL	1 pM/µL	24	616	
UGP-R	TCGGACTTCACTAGCAGCAAATCA		10 pM/µL	1 pM/µL	24		
URA3-F	AGCGAATTGTTGAAGTTGGTTGA	orotidine-5′-phosphate decarboxylase	10 pM/µL	1 pM/µL	23	602	
URA3-R	AATTCGGTTGTAAGATGATGTTGC		10 pM/µL	1 pM/µL	24		

FKS-F—forward primer for the *FKS* gene, FKS-R—reverse primer for the *FKS* gene, LEU2-F—forward primer for the *LEU2* gene, LEU2-R—reverse primer for the *LEU2* gene, NMT1-F—forward primer for the *NMT1* gene, NMT1-R—reverse primer for the *NMT1* gene, TRP1-F—forward primer for the *TRP1* gene, TRP1-R—reverse primer for the *TRP1* gene, UGP1-F—forward primer for the *UGP1* gene, UGP-R—reverse primer for the *UGP1* gene, URA3-F—forward primer for the *URA3* gene, URA3-R—reverse primer for the *URA3* gene.

## Data Availability

Sequences for MLST typing are available from https://pubmlst.org/organisms/candida-glabrata, accessed on 15 March 2025.

## Supplementary Materials

The following supporting information can be downloaded at: https://www.mdpi.com/article/10.3390/ijms26094407/s1, Figure S1: A phylogenetic tree based on the Maximum Likelihood method showing relationships among 30 *N. glabratus* isolates that were MLS typed in this study.; Table S1: Drug susceptibility results; Table S2. Allele sequences characteristics for each locus of the sequence type 212 (ST212); Table S3. Allele sequences characteristics for each locus of the sequence type 213 (ST213); Table S4. Allele sequences characteristics for each locus of the sequence type 214 (ST214).

## Abbreviations

The following abbreviations are used in this manuscript:

Chr	chromosome
MALDI ToF MS	matrix-assisted laser desorption/ionization time-of-flight mass spectrometry
ITS	internal transcribed spacer
MLST	multilocus sequence typing
ST	sequence type
STs	sequence types
*FKS*	1,3-beta-glucan synthase
*LEU2*	3-isopropylmalate dehydrogenase
*NMT1*	myristoyl-CoA protein N-myristoyltransferase
*TRP1*	phosphoribosylanthranilate isomerase
*UGP1*	UTP-glucose-1-phosphate uridylyltransferase
*URA3*	orotidine-5′-phosphate decarboxylase
ID	identification number in the PubMLST database
AB	amphotericin B
FZ	fluconazole
IZ	itraconazole
VOR	voriconazole
PZ	posaconazole
AND	anidulafungin
MF	micafungin
CAS	caspofungin
FC	5-fluorocytosine
MIC	minimal inhibitory concentration [mg/mL]
Interpr.	interpretation
S	susceptible
R	resistant
I	value between the S and the R breakpoint;
IE	insufficient evidence that the organism is a good target for therapy with the agent
NA	not available.
WT	wildtype
non-WT	non-wildtype
ECOFF	Epidemiological Cut-Off Value
EUCAST	The European Committee on Antimicrobial Susceptibility Testing
WGS	whole genome sequencing
NGS	next-generation sequencing
