# Supplementary material for: Nakaseomyces glabratus (Candida glabrata) MLST Genotypes in Central Poland
Source: Int J Mol Sci. 2025 May 6;26(9):4407. doi: 10.3390/ijms26094407 (PMC12072302; doi:10.3390/ijms26094407)
Supplement: Supplementary file 1 [file ijms-26-04407-s001.zip › ijms-3585092-supplementary.pdf]

Table S1. Drug susceptibility results.

ID	ST	AB		FZ		IZ		VOR		PZ		AND		MF		CAS		FC	
		MIC	Interpr.	MIC	Interpr.	MIC	Interpr.	MIC	Interpr.	MIC	Interpr.	MIC	Interpr.	MIC	Interpr.	MIC	Interpr.	MIC	Interpr.
1383	**212**	0.12	S	8.0	I	0.250	IE	0.250	IE	1.000	IE	0.060	S	0.015	S	0.060	S	0.060	NA
1384	**212**	0.50	S	16.0	I	1.000	IE	0.500	IE	2.000	IE	0.030	S	0.015	S	0.030	S	0.060	NA
1385	**213**	0.50	S	16.0	I	0.500	IE	0.500	IE	2.000	IE	0.030	S	0.060	**R**	0.030	**R**	0.060	NA
1386	**214**	0.50	S	8.0	I	0.500	IE	0.250	IE	0.500	IE	0.060	S	0.015	S	0.250	S	0.060	NA
1914	3	0.50	S	4.0	I	0.120	IE	0.060	IE	0.500	IE	0.030	S	0.008	S	0.030	S	0.060	NA
1915	3	0.50	S	4.0	I	0.250	IE	0.120	IE	0.500	IE	0.030	S	0.015	S	0.030	S	0.060	NA
1916	16	2.00	**R**	16.0	I	1.000	IE	1.000	IE	4.000	IE	0.030	S	0.015	S	0.030	S	0.060	NA
1917	7	1.00	S	4.0	I	0.250	IE	0.060	IE	0.500	IE	0.030	S	0.015	S	0.030	S	0.060	NA
1918	22	0.50	S	4.0	I	0.250	IE	0.060	IE	0.500	IE	0.030	S	0.015	S	0.030	S	0.060	NA
1919	6	0.12	S	8.0	I	0.500	IE	0.120	IE	1.000	IE	0.060	S	0.008	S	0.060	S	0.060	NA
1920	22	0.25	S	4.0	I	0.250	IE	0.120	IE	0.500	IE	0.030	S	0.008	S	0.030	S	0.060	NA
1921	49	0.50	S	8.0	I	0.500	IE	0.250	IE	1.000	IE	0.060	S	0.015	S	0.120	S	0.060	NA
1922	22	0.25	S	8.0	I	1.000	IE	0.250	IE	1.000	IE	0.060	S	0.030	S	0.250	S	0.060	NA
1923	76	1.00	S	8.0	I	0.500	IE	0.120	IE	0.500	IE	0.030	S	0.015	S	0.060	S	0.060	NA
1924	3	0.12	S	4.0	I	0.250	IE	0.060	IE	0.500	IE	0.060	S	0.015	S	0.060	S	0.060	NA
1925	3	0.50	S	32.0	**R**	1.000	IE	0.500	IE	2.000	IE	0.060	S	0.008	S	0.120	S	0.060	NA
1926	3	1.00	S	64.0	**R**	16.000	IE	2.000	IE	8.000	IE	0.030	S	0.008	S	0.030	S	0.060	NA
1927	7	1.00	S	32.0	**R**	1.000	IE	0.250	IE	1.000	IE	0.030	S	0.015	S	0.120	S	0.060	NA
1928	10	0.50	S	32.0	**R**	0.500	IE	0.250	IE	2.000	IE	0.060	S	0.015	S	0.060	S	0.060	NA
1929	10	0.25	S	8.0	I	0.500	IE	0.250	IE	0.500	IE	0.250	**R**	0.015	S	0.250	**R**	0.060	NA
1930	6	0.12	S	8.0	I	0.500	IE	0.250	IE	1.000	IE	0.060	S	0.015	S	0.120	S	0.060	NA
1931	6	1.00	S	16.0	I	1.000	IE	0.500	IE	2.000	IE	0.060	S	0.015	S	0.120	S	0.060	NA
1932	6	0.50	S	4.0	I	0.500	IE	0.250	IE	0.500	IE	0.030	S	0.030	S	0.060	S	0.060	NA
1933	10	0.12	S	2.0	I	0.500	IE	0.250	IE	0.250	IE	0.060	S	0.030	S	0.250	S	0.060	NA
1934	45	0.12	S	2.0	I	0.015	IE	0.008	IE	0.500	IE	0.015	S	0.008	S	0.060	S	0.060	NA
1935	203	0.50	S	16.0	I	0.250	IE	0.060	IE	0.250	IE	0.015	S	0.030	S	0.060	S	0.060	NA
1936	2	0.12	S	2.0	I	0.120	IE	0.250	IE	0.500	IE	0.015	S	0.008	S	0.030	S	0.060	NA

1937	2	0.50	S	16.0	I	0.500	IE	0.500	IE	2.000	IE	0.030	S	0.015	S	0.060	S	0.060	NA
1938	55	0.12	S	8.0	I	16.000	IE	0.125	IE	3.000	IE	0.015	S	0.008	S	0.125	S	0.060	NA
1939	3	0.12	S	32.0	R	0.500	IE	0.250	IE	0.500	IE	0.015	S	0.015	S	0.120	S	0.060	NA

ID - identification number in the PubMLST database, ST – sequence type; bolded are the new STs identified in this study, AB - amphotericin B, FZ - fluconazole, IZ – itraconazole, VOR – voriconazole, PZ – posaconazole, AND – anidulafungin, MF – micafungin, CAS – caspofungin, FC - 5-flucytosine, MIC- minimal inhibitory concentration [mg/mL], Interpr. -interpretation, S – susceptible, R – resistant, I - value between the S and the R breakpoint; IE – insufficient evidence that the organism is a good target for therapy with the agent [47], NA – not available.

Table S2. Allele sequences characteristics for each locus of the sequence type 212 (ST212).

Locus	Sequence characteristics for allele type	Allelic variant
FKS	CGGCTACTACGACTCGTACTATAACAACCAGATGAACGCTGGTGTTGGCAATGGGTTGGGACCTGACCAGACTAATTTTTCAGATTTTAGCAGCTACGGACCA<br>CCTCCATTTCAAAATAACCAAGCTAATTATACACCATCCCAACTAAGTTATAGCAACAATGGGATGGGCAGCAACGGTATGAATATGTCTGGTTCGTCAACTC<br>CAGTATACGGTAACTACGATCCGAACGCTATTGCGATGACATTACCAAACGACCCATACCCAGCTTGGACCGCTGATCCACAAAGTCCCGTTTCCATTGAAC<br>AAATCGAAGACGTCTTCATCGATCTAACCAACAAATTTGGTTTCCAAAGAGACTCTATGAGAAACATCTTCGACCTGTTTATGACTTTATTGGACTCTAGAAC<br>ATCCAGAATGTCTCCAGACCAAGCATTGCTATCTGTCCACGCTGATTATATTGGTGGTGACACTGCTAACTACAAGAAATGGTATTTCGCAGCTCAGCTTGAT<br>ATGGATGATGAAGTTGGTTTCAGAAACATGAACTTGGGTAAGTTATCAAGAAAGGCAAGGAAAGCTAAGAAGAA	1
LEU2	ATGGCTGTGACCAAGACAATTGTAGTTCTACCAGGTGACCATGTTGGTCAAGAAATCACTGAAGAGGCCATTAAAGTTTTAAATGCTATTCAGGAATGTCGTC<br>CAGACAAGGTCAATTTCAAGTTTGAGCATCATTTGATCGGTGGTGCTGCAATTGATGCCACTGGTGTTCCATTGCCAGACGAAGCTTTGGAGGCCTCTAAGAA<br>AGCTGATGCCGTGCTTCTGGGTGCTGTTGGTGGTCCAAAATGGGGTACTGGCGCTGTCAGACCAGAACAAGGTCTTTTGAAAATCCGTAAGGAGTTGCAATTG<br>TATGCTAATCTAAGACCATGTAATTTTGCATCTGATTCCTTACTAGATCTATCCCCATTGAAGCCTGAAATTGCAAGAGGTACAGATTTTGTCGTTGTTAGAGA<br>ACTAGTGGGTGGTATTTATTTCGGGGAGAGAAAAGAAGATGAAGGTGATGGTGTCGCCTGGGATAGCGAAAAGTATTCTGTGCCTGAAGTTCAAAGA	23
NMT1	CACAGACCGTTGAACTGGGACAAGCTATATGAGGTCCAGTTCACTGACCTGCCTCCAAACGCCACGAAGGCGGAGATGGTGGCCAAGTACACGCTTCCAAA<br>GGCAACGAAGACCGCCGGTCTGAGAGAGTTGAGACTCGAGGATGTAGACCAGGCCCTGGCACTGTTCAACAGGTACCAGTCCCGGTTTGATATCGTCCAGG<br>AGTTCACCAAGGAAGAGTTCATACATTGGTTTATTAACGACAAGAACGTTGTGGAACAGGACAAGAGGGTTGTATTCTCCTATGTTGTTGAGAGCGAGGGCA<br>AGGTAACAGACTTCTTCTCTTTCTACTCACTGCCATTCACAATCTTGAACAACTCCCGCTACAAGGACCTGGGTATCGGTTACTTATACTACTACGCCTCGGAT<br>GCCGACTTCAAGTTCGAGGACAGGTTCGACAAAGAAGGTACATCGCTCCTAAAGCAAAGACTCTCCACGTTGGTGCAGGACGCATGTATCATCGCGGCACA<br>GAACAAGATGGACGTCTTCAACGCTTTGTCCTCCCAGGATAACACACTGTTCCTGGAAGACCTGAAGTTTGGTCCAGGTGACGGTTCCTGAACTTC	2
TRP1	ATGTCATTTGATTCGTTACTCGACAAGAATGATAAGCTGGTAAAAGTTTGCGGGATTCAAACCGTCGAGGCTGCCGAAACTGCGCTTCAAGCGGGCGCTGATT<br>TGATAGGGATCATATGTGTCCCCAACAGGAAGCGGACTATCGAGAGCGCTGTGGCTCGTGAAATATCCAAATTGATTCACAAATCAGATACTACAAAGCTGG<br>TGGGGGTGTTCAGGAATCAATCTGTTGAGGACGTACATCGGCTTTCTGAGGAATATGACCTTGACATAATCCAATTACATGGTGATGAATCATGGCCAGAGTA<br>CTATAACGTCATTAAGAAACCAATAATCAAAAGAGTCATATTCCCTAGAGATGTCGATGTTGTAACACAAGTGTGTCAAAGAAAACCCTTGGTATGTCTACC<br>ATTGTTC	1
UGP1	GAATTAGATCTTTCAACCAATCCAGATTCCCTCGTGTTTACAAGGACTCCATGCTGCCTGTCCCAGAAACTTACAACGATCCAAAGGACGCTTGGTACCCACC<br>TGGCCATGGTGACTTGTTCGAATCCCTACACGCTTCCGGTGAATTGGACGCTTTAATCGCACAAGGCAGAGAAATCTTATTCGTATCCAACGGTGACAACTTG<br>GGTGCCACTGTCGACCTAAAGATCCTAAACCACATGATCGAAACAGGTGCCGAATACATCATGGAATTGACCGATAAGACCAGAGCCGATGTTAAAGGTGG<br>TACTTTGATCTCTTACGATGGCCAAGTCCGTCTATTGGAAGTCGCCCAAGTTCCAAAGGAGCACATTGATGAATTCAAGAACATCAGAAAATTCACCAACTTC<br>AACACCAACAATTTGTGGATCAACTTGAAGGCTGTTAAGAGATTAGTGGAGTCCAGCGCTTTGGAAATGGAGATTATTCCAAACCAAAAGACCATCACCAGA<br>GGTGGTCAAGAAATCAACGTTCTACAGCTAGAAACCGCCTGTGGTGCCGCCATCAGACACTTTAGCGGTGCTCACGGTGTTGTCGTCCCAAGATCAAGATTCT	1

URA3	TCTATCAGACTTCTCCTTCGAGAACACTGTTAAGCCATTGAAAGAAATGGCAGCCAAGCACAACTTCTTGATTTTTGAAGATAGAAAATTTGCAGACATTGGT<br>AATACTGTTAAGTTGCAATACACATCAGGTGTTTACAAAATTGCAGAATGGGCTGATATCACCAACGCTCATGGTGTTACCGGCCAAGGTATCGTCACTGGTT<br>TAAAGCAAGGTGCTGAGGAAACCACCAATGAACCTAGAGGGCTTTTAATGCTTGCAGAGTTGTCATCTAAGGGCTCTTTAGCTCATGGTGAGTACACTAAAG<br>GCACTGTAGATATTGCTAAATCTGATAAGGACTTTGTCATTGGATTCATTGCTCAAAAGGATATGGGTGGTAGAGACGAGGGCTTTGACTGGCTAATAATGAC<br>TCCTGGTGTCGGTCTAGATGATAAAGGCGATGCACTTGGTCAACAGTACAGAACTGTTGATGAGGTCTTTTCAACCGGTACAGATATTATCATCGTTGGCAGA<br>GGCCTATTCGCCAAGGGCAGAGATCCAAAGACTGAAGGTGAACGTTACAGAAAAGCCGGCTGGGATGCTTACTTGAAAAGAATAGG	1

Table S3. Allele sequences characteristics for each locus of the sequence type 213 (ST213).

Locus	Sequence characteristics for allele type	Allelic variant
FKS	CGGCTACTACGACTCGTACTATAACAACCAGATGAACGCTGGTGTTGGCAATGGGTTGGGACCTGACCAGACTAATTTTTCAGATTTTAGCAGCTACGGACC<br>ACCTCCATTTCAAAATAACCAAGCTAATTATACACCATCCCAACTAAGTTATAGCAACAATGGGATGGGCAGCAACGGTATGAATATGTCTGGTTCGTCAA<br>CTCCAGTATACGGTAACTACGATCCGAACGCTATTGCGATGACTTTACCAAACGACCCATACCCAGCTTGGACCGCTGATCCACAAAGTCCCGTTTCCATTG<br>AACAAATCGAAGACGTCTTCATCGATCTAACCAACAAATTTGGTTTCCAAAGAGACTCTATGAGAAACATCTTCGACCTGTTTATGACTTTATTGGACTCTA<br>GAACATCCAGAATGTCTCCAGACCAAGCATTGCTATCTGTCCACGCTGATTATATTGGTGGTGACACTGCTAACTACAAGAAATGGTATTTCGCAGCTCAGC<br>TTGATATGGATGATGAAGTTGGTTTCAGAAACATGAATTTGGGTAAGTTATCAAGAAAGGCAAGGAAAGCTAAGAAGAA	20
LEU2	ATGGCTGTGACCAAGACAATTGTAGTTCTACCAGGTGACCATGTTGGTCAAGAAATCACTGAAGAGGCCATTAAAGTTTTAAATGCTATTCAGGAATGTCGT<br>CCAGACAAGGTCAATTTCAAGTTTGAGCATCATTTGATCGGTGGTGCTGCAATTGATGCCACTGGTGTTCCATTACCAGACGAAGCTTTGGAGGCCTCTAAG<br>AAAGCTGATGCCGTGCTTTTGGGTGCTGTTGGTGGTCCAAAATGGGGTACTGGCGCTGTCAGACCAGAACAAGGTCTTTTGAAAATCCGTAAGGAGTTGCA<br>ATTGTATGCTAATCTAAGACCATGTAATTTTGCATCTGATTCCTTACTAGATCTATCGCCATTGAAGCCTGAAATTGCAAGAGGTACAGATTTCGTCGTTGTT<br>AGAGAACTAGTGGGTGGTATTTATTTCGGGGAGAGAAAAGAAGATGAAGGTGATGGTGTCGCCTGGGATAGCGAAAAGTATTCTGTGCCTGAAGTTCAAA<br>GA	13
NMT1	CACAGACCGTTGAACTGGGACAAGCTATATGAAGTCCAGTTCACTGACCTGCCTCCGAACGCCACGAAGGCGGAGATGGTGGCCAAGTACACGCTCCCAA<br>AGGCAACGAAGACCGCCGGTCTGAGAGAGTTGAGACTCGAGGATGTAGACCAGGCCCTGGCACTGTTCAACAGGTACCAGTCCCGGTTTGATATCGTCCA<br>GGAGTTCACCAGGGAAGAGTTCATACATTGGTTTATTAACGACAAGAACGTTGTGGAACAGGACAAAAGGGTTGTATTCTCCTATGTTGTTGAGAGCGAGG<br>GCAAGATAACAGACTTCTTCTCTTTCTACTCACTGCCATTCACAATCTTGAACAACTCCCGCTACAAGGACCTGGGTATCGGTTACTTATACTACTACGCCTC<br>GGATGCCGACTTCAAGTTCGAGGACAGGTTCGACAAAGAAGGTACATCGCTCCTAAAGCAAAGACTCTCCACGTTGGTGCAGGACGCATGTATCATCGCG<br>GCACAGAACAAGATGGACGTCTTCAACGCTTTGTCCTTCCAGGATAACACACTGTTCCTGGAAGACCTGAAGTTTGGTCCAGGTGACGGGTTCCTGAACTTC	21
TRP1	ATGTCATTTGATTCGTTACTCGACAAGAATAATAAGCTGGTAAAAGTTTGCGGGATTCAAACCGTCGAGGCTGCCGAAACTGCGCTTCAAGCAGGCGCTGA<br>TTTGATAGGGATCATATGTGTCCCCAACAGGAAGCGGACTATCGAGAGCGCTGTGGCTCGTGAAATATCCAAATTGATTCACAAATCAGATACTACAAAGC<br>TGGTGGGGGTGTTCAGGAATCAATCTGTTGAGGACGTACATCGGCTTTCTGAGGAATATGACCTTGACATAATCCAATTACATGGTGATGAATCATGGCCAG<br>AGTACTATAACGTCATTAAGAAACCAATAATCAAAAGAGTCATATTCCCTAGAGATGTCGATGTTGTAACACAAGTGTGTCAAAGAAAACCCTTGGTATGT<br>CTACCATTGTTC	9
UGP1	GAATTAGATCTTTCAACCAATCCAGATTCCCTCGTGTTTACAAGGACTCCATGCTGCCTGTCCCAGAAACTTACAACGATCCAAAGGACGCTTGGTACCCAC<br>CTGGCCATGGTGACTTGTTCGAATCCCTACACGCTTCCGGTGAATTGGACGCTTTAATCGCACAAGGCAGAGAAATCTTGTTCGTATCCAACGGTGACAACT<br>TGGGTGCCACTGTCGACCTAAAGATCCTAAACCACATGATCGAAACAGGTGCCGAATACATCATGGAATTGACCGATAAGACCAGAGCCGATGTTAAAGG<br>TGGTACTTTGATCTCTTACGATGGCCAAGTCCGTCTATTGGAAGTCGCCCAAGTTCCAAAGGAGCACATTGATGAATTCAAGAACATCAGAAAATTCACCA<br>ACTTCAACACCAACAACTTGTGGATCAACTTGAAGGCTGTTAAGAGATTAGTGGAGTCCAGCGCTTTGGAAATGGAGATTATTCCAAACCAAAAGACCATC<br>ACCAGAGGTGGCCAAGAAATCAACGTTCTACAGCTAGAAACCGCCTGTGGTGCCGCCATCAGACACTTTAGCGGTGCTCACGGTGTTGTCGTCCCAAGATC<br>AAGATTCT	3

URA3	TCTATCAGACTTCTCCTTCGAGAACACTGTTAAGCCATTGAAAGAAATGGCAGCCAAGCACAACTTCTTGATTTTTGAAGATAGAAAATTTGCAGACATTGG TAATACTGTTAAGTTGCAATACACATCAGGTGTTTACAAAATTGCAGAATGGGCTGATATAACCAACGCTCATGGTGTTACCGGCCAAGGTATCGTCACTGG TTTAAAGCAAGGTGCTGAAGAAACCACCAATGAACCTAGAGGGCTTTTAATGCTTGCAGAGTTGTCATCTAAGGGCTCTTTAGCTCATGGTGAGTACACTA AAGGCACTGTAGATATTGCTAAATCTGATAAGGACTTTGTTATTGGATTCATTGCTCAAAAGGATATGGGTGGTAGAGACGAGGGCTTTGACTGGCTAATAA TGACTCCTGGTGTCGGTCTAGATGATAAAGGCGATGCACTTGGTCAACAGTACAGAACTGTTGATGAGGTCTTTTCAACCGGTACAGATATTATCATCGTTG GCAGAGGCCTATTCGCCAAGGGCAGAGATCCAAAGACTGAAGGTGAACGTTACAGAAAAGCCGGCTGGGATGCTTACTTGAAAAGAATAGG	2

Table S4. Allele sequences characteristics for each locus of the sequence type 214 (ST214).

Locus	Sequence characteristics for allele type	Allelic variant
FKS	CGGCTACTACGACTCGTACTATAACAACCAGATGAACGCTGGTGTTGGCAATGGGTTGGGACCTGACCAGACTAATTTTTCAGATTTAGCAGCTACGGACCA CCTCCATTTCAAAATAACCAAGCTAATTATACACCATCCCAACTAAGTTATAGCAACAATGGGATGGGCAGCAACGGTATGAACATGTCTGGTTCGTCAACTC CAGTATACGGTAACTACGATCCGAACGCTATTGCGATGACATTACCAAACGACCCATACCCAGCTTGGACCGCTGATCCACAAAGTCCCGTTTCCATTGAAC AAATCGAAGACGTCTTCATCGATCTAACCAACAAATTTGGTTTCCAAAGAGACTCTATGAGAAACATCTTCGACCTGTTTATGACTTTATTGGACTCTAGAAC ATCCAGAATGTCTCCAGACCAAGCATTGCTATCTGTCCACGCTGATTATATTGGTGGTGACACTGCTAACTACAAGAAATGGTATTTCGCAGCTCAGCTTGAT ATGGATGATGAAGTTGGTTTCAGAAACATGAATTTGGGTAAGTTATCAAGAAAAGCAAGGAAAGCTAAGAAGAA	3
LEU2	ATGGCTGTGACCAAGACAATTGTAGTTCTACCAGGTGACCATGTTGGTCAAGAAATCACTGAAGAGGCCATTAAAGTTTTAAATGCTATTCAGGAATGTCGTC CAGACAAGGTCAATTTCAAGTTTGAGCATCATTTGATCGGTGGTGCTGCAATTGATGCCACTGGTGTTCCATTGCCAGACGAAGCTTTGGAGGCCTCTAAGAA AGCTGATGCCGTGCTTCTGGGTGCTGTTGGTGGTCCAAAATGGGGTACTGGCGCTGTCAGACCAGAACAAGGTCTTTTGAAAATCCGTAAGGAGTTGCAATTG TATGCTAATCTAAGACCATGTAATTTTGCATCTGATTCCTTACTAGATCTATCGCCATTGAAGCCTGAAATTGCAAGAGGTACAGATTTTGTCGTTGTTAGAGA ACTAGTGGGTGGTATTTATTTCGGGGAGAGAAAAGAAGATGAAGGTGATGGTGTCGCCTGGGATAGCGAAAAGTATTCTGTGCCTGAAGTTCAAAGA	21
NMT1	CACAGACCGTTAAACTGGGACAAGCTATATGAAGTCCAGTTCACTGACCTGCCTCCGAACGCCACGAAGGCGGAGATGGTGGCCAAGTACACGCTCCCAAA GGCAACGAAGACCGCCGGTCTGAGAGAGTTGAGACTCGAGGATGTAGACCAGGCCCTGGCATTGTTCAACAGGTACCAGTCCCGGTTTGATATCGTCCAGGA GTTCACCAGGGAAGAGTTCATACATTGGTTTATTAACGACAAGAACGTTGTGGAACAGGACAAGAGGGTTGTATTCTCCTATGTTGTTGAGAGCGAGGGCAA GATAACAGACTTCTTCTCTTTCTACTCACTGCCATTCACAATCTTGAATAACTCCCGCTACAAGGACCTGGGTATCGGTTACTTATACTACTACGCCTCGGATG CCGACTTCAAGTTCGAGGACAGGTTCGACAAAGAAGGTACATCGCTCCTAAAGCAAAGACTCTCCACGTTGGTGCAGGACGCATGTATCATCGCGGCACAG AACAAGATGGACGTCTTCAACGCTTTGTCCTCCCAGGATAACACACTGTTCCTGGAAGACCTGAAGTTTGGTCCAGGTGACGGGTTCCTGAACTTC	26
TRP1	ATGTCATTTGATTCGTTACTCGACAAGAATGATAAGCTGGTAAAAGTTTGCGGGATTCAAACCGTCGAGGCTGCCGAAACTGCGCTTCAAGCAGGCGCTGATT TGATAGGGATCATATGTGTCCCCAACAGGAAGCGGACTATCGAGGGCGCTGTGGCTCGTGAAATATCCAAATTGATTCACAAATCAGATACTACAAAGCTGG TGGGGGTGTTCAGGAATCAATCTGTTGAGGACGTACATAGGCTTTCTGAGGAATATGACCTTGACATAATCCAATTACATGGTGATGAATCATGGCCAGAGTA CTATAACGTCATTAAGAAACCAATAATCAAAAGAGTCATATTCCCTAGAGATGTCGATGTTGTAACACAAGTGTGTCAAAGAAAACCCTTGGTATGTCTACC ATTGTTC	13
UGP1	GAATTAGATCTTTCAACCAATCCAGATTCCCTCGTGTTTACAAGGACTCCATGCTGCCTGTCCCAGAAACTTACAACGATCCAAAGGACGCTTGGTACCCACC TGGCCATGGTGACTTGTTCGAATCCCTACACGCTTCCGGTGAATTGGACGCTTTAATCGCACAAGGCAGAGAAATCTTGTTTGTATCCAACGGTGACAACTTG GGTGCCACTGTCGACCTAAAGATCCTAAACCACATGATCGAAACAGGTGCCGAATACATCATGGAATTGACCGATAAGACCAGAGCCGATGTTAAAGGTGG TACTTTGATCTCTTACGATGGCCAAGTCCGTCTATTGGAAGTCGCCCAAGTTCCAAAGGAGCACATTGATGAATTCAAGAACATCAGAAAATTCACCAACTTC AACACCAACAACTTGTGGATCAACTTGAAGGCTGTTAAGAGATTAGTGGAGTCCAGCGCTTTGGAAATGGAGATTATTCCAAACCAAAAGACCATCACCAGA GGTGGCCAAGAAATCAACGTTCTACAGCTAGAAACCGCCTGTGGTGCCGCCATCAGACACTTTAGCGGTGCTCACGGTGTTGTCGTCCCAAGATCAAGATTCT	17
URA3	TCTATCAGACTTCTCCTTCGAGAACACTGTTAAGCCATTGAAAGAAATGGCAGCCAAGCACAACTTCTTGATTTTTGAAGATAGAAAATTTGCAGACATTGGT AATACTGTTAAGTTGCAATACACATCAGGTGTTTACAAAATTGCAGAATGGGCTGATATCACCAACGCTCATGGTGTTACCGGCCAAGGTATCGTCACTGGTT	4

	TAAAGCAAGGTGCTGAGGAAACCACCAATGAACCTAGAGGGCTTTTAATGCTTGCAGAGTTGTCATCTAAGGGCTCTTTAGCTCATGGTGAGTACACTAAAG<br>GCACTGTAGATATTGCTAAATCTGATAAGGACTTTGTCATTGGATTCATTGCTCAAAAGGATATGGGTGGTAGAGACGAGGGCTTTGACTGGCTAATAATGAC<br>TCCTGGTGTCGGTCTAGATGATAAAGGCGATGCACTTGGTCAACAGTACAGAACTGTTGATGAGGTCTTTTCAACCGGTACAGATATTATCATCGTTGGCAGA<br>GGCCTATTCGCCAAGGGCAGAGATCCAAAGACTGAAGGTGAACGATACAGAAAAGCCGGCTGGGATGCTTACTTGAAAAGAATAGG	

ID – identification number in the PubMLST database, ST – sequence type; *FKS* - 1,3-beta-glucan synthase , LEU2 - 3-isopropylmalate dehydrogenase, *NMT1* - myristoyl-CoA, protein N-myristoyltransferase, *TRP1* - phosphoribosylanthranilate isomerase, *UGP1* - UTP-glucose-1-phosphate uridylyltransferase, *URA3* - orotidine-5'-phosphate decarboxylase.

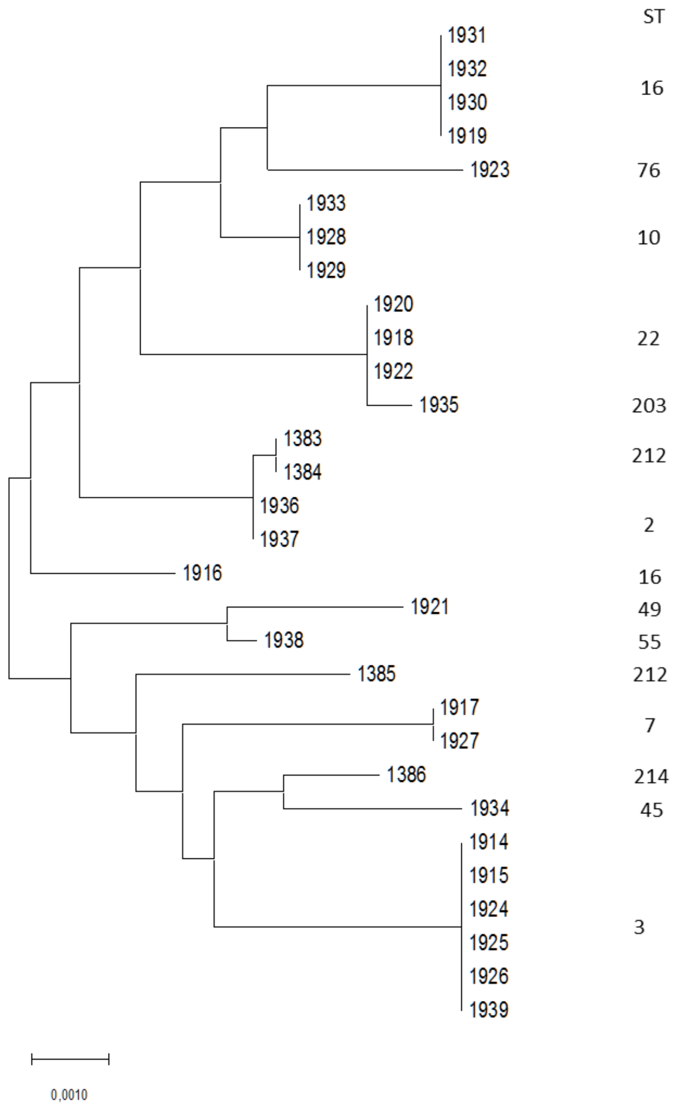

Figure S1. A phylogenetic tree based on the Maximum Likelihood method showing relationships among 30 *N. glabratus* isolates that were MLS typed in this study. The number at the end of a branch indicates the isolate ID number in the MLST database.
